# The psychotherapeutic care of refugees in Europe: treatment needs, delivery reality and recommendations for action

**DOI:** 10.1080/20008198.2018.1476436

**Published:** 2018-06-25

**Authors:** Dietrich Munz, Nikolaus Melcop

**Affiliations:** Bundes Psychotherapeuten Kammer (BPtK), Berlin, Germany

**Keywords:** Refugees, trauma, mental healthcare, psychotherapy, refugiados, trauma, cuidado de la salud mental, Psicoterapia, 难民, 创伤, 精神保健, 心理治疗

## Abstract

The special issue of the *European Journal of Psychotraumatology* released on 7 November 2017 focused on traumatized refugees and on the mental health burden, screening instruments and interventions in different groups of refugees. This contribution takes up this discussion on the needs and challenges for mental healthcare of traumatized refugees from the point of view of the practitioners. It reports on the findings of a survey on the treatment situation and the delivery reality of healthcare for refugees in 14 European countries, identifies treatment gaps, and sets recommendations for action at the political and therapeutic levels. The survey was conducted by the Federal Chamber of Psychotherapists with the assistance of the Network for Psychotherapeutic Care in Europe. The findings underline the need for appropriate mental healthcare for this population.

## Abstract (es)

El número especial del Journal Europeo de Psicotraumatología publicado el 7 de Noviembre de 2017 se focaliza en refugiados que han sufrido trauma, la carga de salud mental, instrumentos de tamizaje e intervenciones en diferentes grupos de refugiados. Su contribución adicional es que pone énfasis en el punto de vista de los profesionales de salud respecto a las necesidades y desafíos para el cuidado de la salud mental de los refugiados traumatizados. Informa sobre los hallazgos de una encuesta sobre la situación de tratamiento y realidad de entrega del cuidado de salud para refugiados en 14 países Europeos, identifica las brechas de tratamiento y establece recomendaciones para la acción a un nivel político y terapéutico. La encuesta fue conducida por la Cámara Federal de Psicoterapeutas (BPtk) con la asistencia de la Red para el Cuidado Psicoterapéutico en Europa (NPCE). Los hallazgos refuerzan la necesidad de un cuidado de salud mental apropiado para esta población.

## Abstract (zh)

《欧洲心理治疗学杂志》2017年11月7日发布的特刊专注于受创伤的难民，心理健康负担，筛查工具和不同难民群体的干预。 其中的贡献包括从实践者的角度讨论了关于创伤难民的精神卫生保健需求和挑战。 它报告了一项关于14个欧洲国家的难民医疗状况和医疗服务提供现况的调查结果，指出了治疗差距并从政治和治疗层面提出了实践建议。 该调查由联邦心理治疗师协会（BPtK）在欧洲心理治疗护理联盟（NPCE）的协助下进行。 这些发现强调了这一人群需要适当的精神卫生保健。

## 1. Introduction

## 

In 2016, around 1.2 million people sought protection within the European Union (EU) from war and persecution in their home countries. Germany was the first country of asylum for an above-average number of these people (8,789 per million inhabitants), but the corresponding proportionate figures for Greece (4,625), Austria (4,587), Malta (3,989), Cyprus (3,350), Hungary (2,870) and Bulgaria (2,655) also exceeded the EU-wide average of 2,360 asylum seekers per million inhabitants.

Christine Knaevelsrud, Nadine Stammel and Miranda Olff (2017) refer in their editorial to the international studies on the prevalence of mental health problems among refugees. Refugees are people who are forced to leave their home country either temporarily or permanently because of political repression, war or life-threatening hardship. Legally, there is a distinction between asylum seekers and refugees who have successfully completed their asylum recognition procedure. Although a significant percentage of these refugees are mentally ill and in need of treatment, currently, too few of them are receiving support and professional help (Sijbrandij et al., 2017). Our stocktaking process makes it possible to describe and better assess the situation in different countries.

## 2. Mental stress suffered by refugees

In their homelands, as well as while on the run after fleeing them, many refugees have been subjected to extremely traumatic experiences, including war, forced migration, physical and sexual violence, torture, hunger, thirst and cold. Often, they feared for their lives or those of their loved ones, or even witnessed others die. These psychologically disturbing events cause massive anxiety, despair, a sense of helplessness, a loss of confidence in the future and mistrustfulness.

The most common mental illness among refugees is post-traumatic stress disorder (PTSD), with symptoms including flashbacks, overexcitement, avoidance of situations and thoughts associated with the trauma, sleep and concentration disorders, and a loss of trust in other people. Yet, other mental disorders can also arise, such as depressive disorders, anxiety disorders, somatic symptom disorders, dissociative disorders and substance dependency. Those affected then struggle to cope with the challenges of integrating into the host society.

## 3. Prevalence of mental disorders among refugees

A structured questionnaire was used to gather data regarding the prevalence of psychological disorders among refugees, the state of treatment and its legal context in host countries, and to identify best-practice examples and areas where action was required. Those asked to complete the survey were experts from institutions that deal with the psychotherapeutic care of refugees, such as psychologists and psychotherapists working in refugee reception centres, researchers at universities and representatives of professional organizations. Members of the European Society for Traumatic Stress Studies (ESTSS) also took part in the survey. Altogether, psychotherapists, psychologists, physicians and health managers from 14 European countries were interviewed. (The report includes results from the EU member countries Austria, Belgium, Cyprus, Germany, Greece, Hungary, Ireland, Italy, the Netherlands, Poland, Portugal, Sweden and the UK, as well as from Switzerland.) In March 2017, a workshop (http://www.npce.eu/index.html) was held at which the surveyed experts discussed the survey’s findings and drew conclusions. A report of the findings was published in October 2017. The full report and the list of experts included can be found at: http://www.npce.eu/mediapool/113/1137650/data/20171006/psychotherapeutic_care_for_refugees_in_europe.pdf.

Not every country has carried out its own investigations. The reported results from recent studies confirm that there are higher prevalences of PTSD, depression and other mental disorders among refugees than among the general population. According to the research, about half of adult refugees and about one-third of refugee children in Germany suffer from a mental illness. The incidence of PTSD is at least 20% (BPtK, 2018).

A study by the Swedish Red Cross in 2016 (Tinghög, Arwidson, Sigvardsdotter, Malm, & Saboonchi, 2016) revealed a PTSD rate of 30.1% among ‘newly-arrived refugees’ (Refugees are designated as such within the first 2 years of their stay). In Switzerland, 23.3% of refugees were diagnosed with PTSD (Heeren et al., 2012). In the Netherlands, Adulkram, determined, inter alia, that even 6–22 years later, refugees still show a higher susceptibility to PTSD than the general population (Ikram & Stronks, 2016).

A long-term study in Italy (Pfarrwaller & Suris, 2012) identified a number of predictors of long-term mental health or illness among young refugees. It found that, even 8–9 years after arrival, there remained a correlation between mental health problems and the number of post-arrival stressful events, experiences of discrimination, and insufficient stability and integration into the host society. Administrative procedures are a further source of stress (Stuart, 2015). A correlation was identified between a long asylum review process and anxiety, as well as depressive and somatoform disorders. There is evidence from the UK that refugees face a higher risk of homelessness and poverty, which can also have a major impact on their mental health (www.gov.uk).

As with physical illness, the question of whether treatment is required – and if so, which one – is dependent upon the severity of the mental illness. The delivery of a differentiated, tiered range of care is therefore necessary. Refugees who are suffering from mild mental health difficulties often recover on their own once they are in a safe environment. Others are helped by psychosocial counselling. Some, however, require a specific treatment, which can range from outpatient psychotherapy to inpatient treatment in a psychiatric clinic. Yet, according to a recent Dutch study, only 20% of refugees with PTSD sought psychotherapeutic assistance, although there are effective treatment options for traumatized and mentally ill refugees (e.g. Acarturk et al., 2015; Stammel et al., 2017). In the course of the 7 year follow-up period, the proportion increased to 54%, but remained relatively low (www.gezondheidsraad.nl). Another study (Laban, Gernaat, Komproe, & De Jong, 2007) in the Netherlands found that only 9% of asylum seekers with mental disorders had visited a mental health institution.

## 4. Treatment needs versus the delivery reality

Providing appropriate psychotherapeutic care to refugees is a challenge for the healthcare systems of the host countries. Although the member states differ in terms of the systems that organize healthcare and the resources available to care for refugees, the requirements with respect to providing adequate care are similar.

The results of the survey show that, in most countries, refugees are only entitled to limited acute care. Long waiting periods often occur and care-giving staff are often not adequately qualified to provide the specialized treatment that refugees require. Further, having the same formal rights as citizens to receive psychotherapeutic care is of no use to refugees if this care has yet to be regulated for citizens (as in Belgium), or if care delivery bottlenecks are occurring or additional fee requirements are in place (as in Austria, Greece and Poland).

There exists considerable friction between the authorities and those providing care. Moreover, specialists have frequently not been specially trained to adequately meet the particular requirements of caring for refugees. In some countries, there are shortages of professionals who are able to evaluate signs of torture, diagnose mental illnesses and provide treatment indications. A major problem is a lack of qualified interpreters and language mediators (including the funding thereof), who are essential to providing any psychotherapeutic care at all to refugees.

It is particularly difficult for refugees to cope with trauma when their future prospects are uncertain and when burdens related to the asylum application process and to housing are added to this uncertainty. Difficult housing conditions, restrictions on family reunification and drawn-out asylum application can pose a risk to those with mental disorders.

Basic care that is limited to the treatment of acute and severe somatic disorders is not enough, as it fails to recognize the consequences of non-treatment and the negative long-term consequences of mental illness, in particular PTSD. The longer a person in need of treatment remains untreated, the greater the risk of a crisis occurring that then necessitates more intensive treatment.

Receiving psychotherapeutic treatment can be unfamiliar to refugees. Indeed, some consider being mentally ill and needing assistance for this to be disgraceful. Most refugees instead attempt to integrate and forget past traumatic events. It is to be expected that the existence of trauma and the need for psychotherapeutic treatment will only become apparent over time.

It is important for a country’s citizens to have more empathy and respect for refugees, and for there to be a greater degree of mutual understanding between the two groups. The relationship they share is not one sided. The therapists, as well as the society as a whole, can also learn important things from the people who seek refuge in their country.

The results of the survey were discussed in the expert workshop and led to recommendations for action at different levels.

## 5. Recommendations for action in terms of health policy legislation

Ȗ Mentally ill refugees are included among the most vulnerable under the EU’s Reception Conditions Directive (Directive 2013/33/EU) and are thus entitled to necessary medical or other assistance. However, this EU directive has largely yet to be implemented at the national level. ‘Essential treatment’, which is often all that refugees are entitled to, focuses on acute physical illnesses and does not adequately take mental illnesses into account.
Ȗ Structures are needed for the early detection of particularly vulnerable refugees. The procedure followed in Sweden is exemplary. There, all arriving refugees are examined with regard to both their physical and mental health. Furthermore, there are instruments that can help to identify mental health problems in refugees (e.g. Kaltenbach, Härdtner, Hermenau, Schauer, & Elbert, 2017).
Ȗ Psychotherapeutic care must be available to all those who need it, both citizens and refugees. The services of interpreters and language mediators, which are essential to the provision of any medical or psychotherapeutic treatment at all, must be financed. The interpreters and language mediators must receive intercultural training, as well as training to translate in the psychotherapeutic context.
Ȗ Legal rights must be practically applied. In many countries, medical and psychotherapeutic treatment of refugees is only carried out through the involvement of non-governmental organizations.
Ȗ For multi-professional care to be reliably provided, a legal framework for the healthcare system must be established, which ensures that refugees with special needs do not fall through gaps in the healthcare delivery system.
Ȗ Asylum application processes must be accelerated to reduce long waiting periods and the psychological insecurity that accompanies them. PTSD must be classified as an obstacle to deportation.
Ȗ More funding must be made available to research.

## 6. Organizational conditions for successful psychotherapeutic work with refugees

Ȗ Best-practice models should be adopted into standard care practice.
Ȗ Ongoing healthcare delivery projects need reliable, long-term financing. This requires more state funding and larger dedicated budgets at both the national and international levels.
Ȗ It is necessary to set up workgroups and centres that pool skills and competencies, coordinate care and close gaps between different services, such as reception centres and outpatient facilities. Psychiatric hospital capacities must also be expanded to meet requirements.
Ȗ In addition to sufficient healthcare, refugees need support in other relevant areas of life such as employment and further education (Zepinic, Bogic, & Priebe, 2012). This can help to strengthen the resilience of refugees (Sleijpen, Heide, Mooren, Boeije, & Kleber, 2013).

## 7. A need for action at the therapeutic level

Ȗ Treating people from other cultures, as well as working with interpreters, can present particular challenges for psychotherapy practitioners. To deal with these challenges, it can be helpful for psychotherapists to gain knowledge and experience in intercultural psychotherapy and practising psychotherapy with the assistance of interpreters, e.g. through training courses.
Ȗ In addition to professional assistance, social support and contact is essential to providing psychotherapeutic care to refugees. This requires a tiered treatment concept that ranges from self-help, support from other trained refugees or migrants, and psychosocial counselling services to psychotherapy and inpatient psychiatric–psychotherapeutic care. Internet-based programmes can be useful in this context. In the case of refugee children, special age-appropriate and culturally sensitive concepts must be employed. All service providers should work together as a multi-professional team.
Ȗ People suffering from trauma need a safe environment. They should therefore never be deported once psychotherapy has begun.
Ȗ Continuity of treatment must be ensured whenever asylum seekers are transferred to other reception centres during treatment or whenever their status changes.
